# Susceptibility to *klebsiella pneumonaie* infection in collaborative cross mice is a complex trait controlled by at least three loci acting at different time points

**DOI:** 10.1186/1471-2164-15-865

**Published:** 2014-10-06

**Authors:** Karin Vered, Caroline Durrant, Richard Mott, Fuad A Iraqi

**Affiliations:** Department of Clinical Microbiology and Immunology, Sackler Faculty of Medicine, Tel Aviv University, Tel Aviv, Israel; Wellcome Trust Centre for Human Genetics, University of Oxford, Oxford, OX3 7BN UK

**Keywords:** Klebsiella pneumoniae, Mouse model, Collaborative cross mice, Host susceptibility, QTL mapping, Candidate genes

## Abstract

**Background:**

*Klebsiella pneumoniae* (Kp) is a bacterium causing severe pneumonia in immunocompromised hosts and is often associated with sepsis. With the rise of antibiotic resistant bacteria, there is a need for new effective and affordable control methods; understanding the genetic architecture of susceptibility to Kp will help in their development. We performed the first quantitative trait locus (QTL) mapping study of host susceptibility to Kp infection in immunocompetent Collaborative Cross mice (CC). We challenged 328 mice from 73 CC lines intraperitoneally with 10^4^ colony forming units of Kp strain K2. Survival and body weight were monitored for 15 days post challenge. 48 of the CC lines were genotyped with 170,000 SNPs, with which we mapped QTLs.

**Results:**

CC lines differed significantly (P < 0.05) in mean survival time, between 1 to 15 days post infection, and broad sense heritability was 0.45. Distinct QTL were mapped at specific time points during the challenge. A QTL on chromosome 4 was found only on day 2 post infection, and QTL on chromosomes 8 and 18, only on day 8. By using the sequence variations of the eight inbred strain founders of the CC to refine QTL localization we identify several candidate genes.

**Conclusion:**

Host susceptibility to Kp is a complex trait, controlled by multiple genetic factors that act sequentially during the course of infection.

**Electronic supplementary material:**

The online version of this article (doi:10.1186/1471-2164-15-865) contains supplementary material, which is available to authorized users.

## Background

*Klebsiella pneumoniae* (Kp) is a gram-negative enteric bacillus that is a common cause of nosocomial pneumonia after surgery, with significant associated morbidity and mortality [1, 2]. With the rise of antibiotic resistance in bacteria, there is a need for alternative, effective and affordable control methods. Mouse models for Kp infection are well established, with a particular focus on the host immune response and the mechanisms induced to aid bacterial clearance [3]. It is well known that common inbred strains of mice often show differences in their genetic predisposition to infectious diseases. Genetic mapping has been used successfully to identify a number of murine loci conferring resistance to pathogenic diseases [4–6]. Consequently, the host response to Kp infection should also be amenable to genetic methods.

Although intercrosses between inbred lines of mice are powerful tools for mapping quantitative trait loci (QTL), with some important exceptions the genes underlying the QTLs remain unknown because the QTL intervals are too broad. Furthermore, because classical laboratory strains of mice originate from just a small sample of founders, they have a remarkably high level of shared ancestry largely contributed by the *M. m. domesticus* subspecies, and contain limited diversity. In contrast, wild-derived inbred strains encompasses genetic variation accumulated over about one million years. Thus each classical laboratory strain differs from the reference C57BL/6 J at about 4 million SNPs, whilst the wild-derived strains CAST/Ej and PWK/PhJ each differ at 17 million SNPs, and WSB/EiJ at 6 million [7].

Recognition of these problems resulted in the construction of the Collaborative Cross (CC), a general-purpose mouse resource to model complex traits [8, 9]. The CC is a population of recombinant inbred lines descended from eight divergent strains of mice, comprising five classical strains descended from *M. m. domesticus* (A/J, C57BL/6 J, 129S1/SvImJ, NOD/LtJ, NZO/HiLtJ), combined with three wild-derived strains CAST/Ei (*M. m. castaneus*), PWK/PhJ (*M. m. musculus*), and WSB/EiJ (*M. m. domesticus*) [8]. Over 35 million SNPs segregate between the CC founders. Wild mice are constantly attacked by pathogens and the specificity and efficiency of their innate mechanisms of defense are under strong selective pressure.

We previously demonstrated the power of the immune-competent CC lines for mapping QTL associated with host susceptibility to infection in a study of susceptibility to *Aspergillus fumigatus* [10]. The high genetic diversity in the CC population meant that we could map QTLs that involved contrasts of alleles segregating between the wild-derived strains, and which would not have been visible in a cross between classical strains. These results suggested that host susceptibility to (Kp) infection should be amenable to investigation in the CC as well. In this study, we map susceptibility to Kp in the CC, and identify QTL that are active in response to infection at distinct time points. Thus we have begun to dissect the underlying genetic and temporal architecture of Kp infection.

## Methods

### Mice

Mice were housed on hardwood chip bedding in open-top cages. They were kept on a 12-hour light/dark cycle and received distilled water and food ad libitum*,* before and during the challenge. All experimental protocols were approved by the Institutional Animal Care and Use Committee at TAU (IACUC), which adheres to Israeli guidelines, which follow the NIH/USA animal care and use protocols.

### Inbred mice

Ten female mice aged eight weeks from each of the mouse strains BALB/CJ, DBA/2 J, C57BL/6 J and C3H/J were purchased from Harlan, Rehovot, Israel and used for testing.

### Collaborative cross mice

Full details of the development of the CC colony are described in [9]. In this study 328 male (144 mice) and female (184 mice) CC mice from 73 lines (average 3–5 mice per line) at inbreeding generations of 8–17 and between 8 to 12 weeks were used.

### Klebsiella pneumonia (Kp) inoculation

Infection experiments with Kp were performed at the small animal facility at Sackler Faculty of Medicine, Tel-Aviv University (TAU), Israel*. Klebsiella pneumoniae* strain K2 was provided by Izthak Ofek, Department of Clinical Microbiology and Immunology, Sackler Faculty of Medicine, Tel-Aviv University in the mid-log phase was obtained by growth in Luria broth (LB) composed of 10% Bacto Tryptone (Difco), 5% yeast extract, and 5% NaCl (pH 7.5) (Difco Laboratories, Detroit, MI) for 18 h at 37°C followed by inoculation into LB media for an additional 4 h. K2 strain used in this study was isolated from human blood with full details is presented in previous reports [11, 12] and was shown to be generally virulent in mice [13].

The Kp concentration in the broth was quantified by comparing absorbance at 595 nm with a standard curve. Bacteria were then diluted with sterile endotoxin-free saline to provide **10**^**4**^ colony forming units (cfu) in a final volume of 0.2 ml. Bacterial numbers were confirmed by colony counts of LB agar plate dilutions after 24-h incubation at 37°C. Animals were injected intraperitoneally (IP) with a final volume of 0.2 ml.

### Kp challenge

Immunocompetent mice were challenged intraperitoneally with 10^4^ cfu of K2 Kp. Clinical assessment of susceptibility to infection during 15 days post challenge was based primarily on survival time. In addition, body weight and rectal body temperature, by a designed thermometer for small animal (NBT New Biotechnology Ltd. Jerusalem, Israel), was measured in survived animals at day 0, 2, 4, 8 and 14. Post-mortem testing for presence of Kp was by plating extracts from mouse tissues on MacConkey agar plates and counting cfu. Tail clips from all CC mice were saved as a DNA source.

### Postmortem testing of CFU Loads

All succumbed and survived mice to infection were tested postmortem for K2 loads by plating extracts from different tissues for growth on LB media which solidified with 1.5% Bacto Agar and CFU counts method.

### Genotyping

Representatives of 48 of the 73 CC lines used in this study had been genotyped previously (at WTCHG Oxford UK and at CISGen, UNC, USA), at 170000 SNPs, as described in [10]. Although the CC lines are not completely inbred, we have shown previously that using genotypes from a single representative from each line is sufficient for QTL mapping purposes.

### Data analysis

Data analysis was performed using the statistical software R (R Development Core Team 2009), including the R package HAPPY.HBREM [14, 15]. Survival data for the CC lines were converted into binary alive/dead phenotypes, one for each day of the trial. These binary phenotypes were analysed for the presence of QTLs by a two-stage process. Firstly, a logistic regression model was used to fit covariates, using the R function glm(), and secondly the residuals from the model were used as the response variable for QTL mapping using linear regression, with the Bayesian random effects model HBREM [15] used to estimate the individual haplotype effects.

Two covariates significantly affected survival, sex and a batch effect. These effects were fitted via a logistic regression model where, for an individual of sex i in batch group j with alive/dead status y_ij_,


Where *π*(*y*_*ij*_) is the probability of being dead, *μ* is the intercept, *α*_*i*_ is the effect of sex, and *γ*_*j*_ is the effect of batch.

The genome of each CC line is a mosaic of the inbred founders, which we reconstructed using a hidden Markov Model (HMM) HAPPY [14] across the genotypes to compute probabilities of descent from the founders, setting the generation parameter to g = 7. In CC line *k* at SNP interval (locus) *L*, the HMM probability of descent from founder strain *s* is denoted by . The presence of a QTL at the locus *L* is tested using a linear regression framework, in which the residual deviance from the mean probability of death *Y*_*k*_ for an individual from line *k*:


where μ is the overall mean (incorporating any effects of batch and sex), and *β*_*s*_ is the effect of founder haplotype *s* at locus *L*.

The presence of a QTL is tested by comparing the fit of the model with that of a simpler submodel in which all the *β*_*s*_ = 0 (the null hypothesis). Significance is reported as the logP, the negative log_10_ of the p-value of the test of the null hypothesis, as computed by the R anova() function. Genomewide significance was estimated by permutation, where the CC line labels were permuted between the phenotypes. The median probability of death across replicates within each CC line was used in the QTL analysis. QTL effect sizes were estimated as the proportion of the log likelihood explained by the locus effects at the QTL.

### Estimation of heritability

Heritability H^2^ was estimated as the proportion of phenotypic variation explained by differences between CC lines in the ANOVA, i.e. *H*^*2*^ *= V*_*g*_*/(V*_*g*_ *+ V*_*e*_*)*. Full details on the heritability and Coefficient of Variation in CC mice were presented in our recent study [16].

### Estimation of QTL confidence intervals

We estimated the CI for each QTL by simulation, using a similar approach as used in [10], to take into account local patterns of linkage disequilibrium.

### Testing sequence variation segregating between the CC founders

We used the merge analysis [17] to test which variants under a QTL peak were compatible with the pattern of action at the QTL. We used the Sanger mouse genomes database [5] of sequence variants.

## Results

### Susceptibility to infection by *Klebsiella pneumonia*

We first compared the responses of four immune competent inbred strains (ten females from each of BALB/CJ, DBA/2 J, C3H/HeJ and C57BL/6 J) and 73 CC lines (3–7 mice per line) to infection with Kp to establish our assay was effective. Postmortem testing confirmed that all mice were infected with high cfu loads, indicating that death was caused by infection. All mice died during the infection, but with heritable variation in survival time (Additional file 1: Table S1). BALB/CJ mice were highly susceptible, DBA/2 J and C3H/HeJ were highly resistant and C57BL/6 J was intermediate. BALB/cJ was significantly different from the three other strains (Additional file 1: Table S1). Mean survival time of BALB/CJ, C57BL/6 J, C3H/J and DBA/2 J was 2 days (s.d = 0.6), 2.8 days (sd = 0.56), 3.8 days (s.d = 1.16), and 4 days (s.d = 1.88), respectively.

Body temperature and weight were shown not to be significantly changed during the infection, and have shown no correlations with survival time post infection, therefore were not presented and fully discussed in this report.

**Figure 1:**
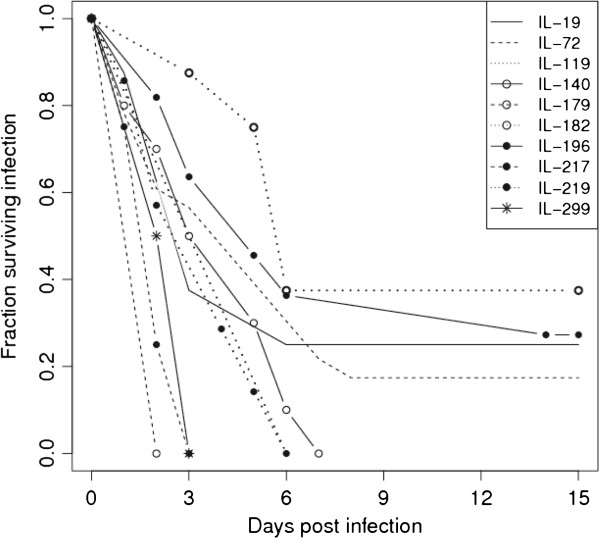
**Survival curves of representative collaborative cross lines after intraperitoneal infection with** ***Klebsiella pneumoniae*** **.**

**Figure 2:**
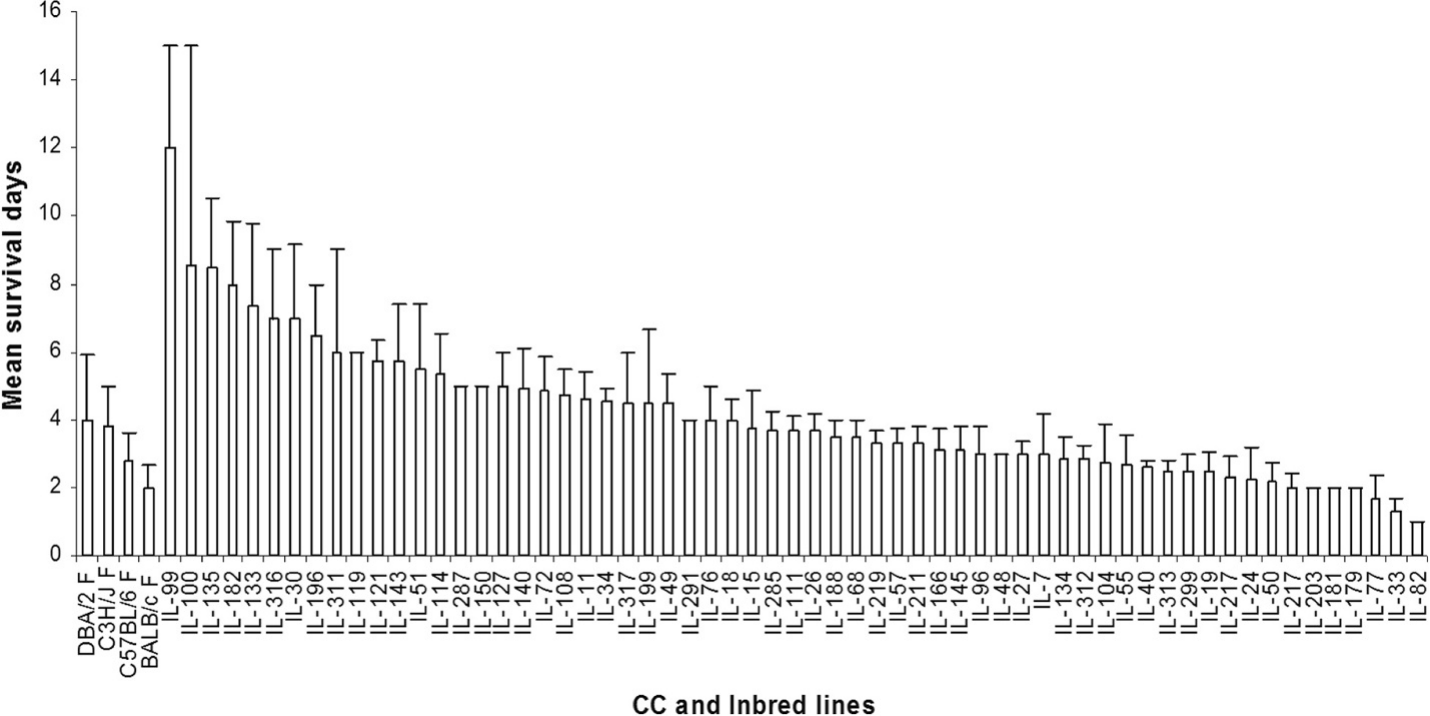
**Mean survival times after infection with** ***Klebsiella pneumonia*** **.** Mice from four classical inbred and representative Collaborative Cross (IL) lines were challenged and average survival times in days were calculated for each line, with standard errors indicated.

The 328 CC mice also responded variably with mean survival time between 1 to 12 days. Differences in survival between the 73 CC lines were highly significant (P < 0.0001). Figure 1 shows the percentage survival of a representative selection of susceptible, intermediate and resistant lines, Figure 2 shows the mean survival time (days) and standard deviation (s.d) of the representative selection CC lines and Additional file 2: Table S2 summarize the mean survival time (days) and standard deviation (s.d) of the entire studied CC lines. Although the variation between lines was highly significant, indicating that the response to infection was heritable, there was also considerable variability within some lines. Mice that survived past the seventh day of infection tended to survive to the end of the experiment, suggesting this was a critical point in the disease progression.

The CC mice were assayed in 10 batches, two of which had different survival times, so we fitted a three-level batch covariate (P = 6.84e-17). The number of inbreeding generations of each CC line had no effect on survival. However, sex was significant (P = 9.66e-05), with females less susceptible than males with mean survival days of 4.86 and 3.71, respectively. No correlation was observed between body weight (BW) and survival time (Pearson R^2^ = -0.07), or between body temperature (BT) and survival time (R^2^ = 0.053).

### QTL mapping

**Figure 3:**
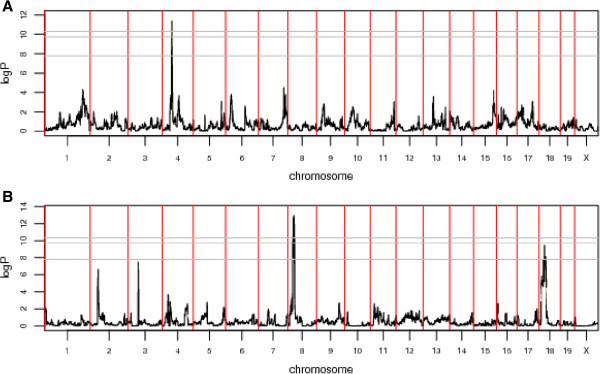
**Genome scans for days with significant QTLs.** Three QTL associated with survival time were detected, on days 2 and 8 after infection with Kp. Experiment-wide thresholds of significance at 50%, 90% and 95% levels are logP = 7.8, 9.7 and 10.3, respectively (e.g. the p% threshold means that in p% of permutations the genome wide maximum logP across all analyses at different time points did not exceed the threshold). **A**. Scan at day 2 post infection showing QTL *Kprl*1 on chromosome 4 at day 2 of infection. **B**. Scan at day 8 post infection showing QTLs, *Kprl*2 and *Kprl*3 on chromosomes 8 and 18.

We used a similar QTL mapping methodology to that previously employed in a study of host susceptibility to Aspergillosis in CC mice [10], except that we tested alive/dead survival status for QTLs at different time points, to identify early- and late-acting QTLs. Figure 3 and Table 1 summarize the three QTLs we found associated with host susceptibility to Kp infection at genomewide E < 0.5 (FDR = 8.3%). These QTL, named ***Kprl1 – Kprl3***, (***K***lebsiella ***p***neumonia ***r***esistant ***l***ocus) were located on chromosomes 4, 8, and 18 respectively, *Kprl*1 and 2 were mapped with 50% confidence intervals (50% CIs) of 0.48 and 0.51 Mb and 95% CIs of 7.03 and 5.44 Mb. Plots of the simulation results for the CIs are in Additional file 3: Figure S1. *Kprl*3 was mapped at 50% genome wide significance (i.e. in 50% of genome scans with permuted CC line designations the genome-wide maximum logP was less than that observed at *Kprl*3), to a 50% interval of 5.85 Mb and a 95% interval of 18.06 Mb. However, the CI simulations around *Kprl*3 showed a different LD pattern focussed on the location of the original QTL, regardless of the location of the simulated locus (see Additional file 3: Figure S1). The two QTLs found on day 8 are clearly correlated. However, they show different patterns of haplotype effects so there does appear to be two different QTLs.

**Figure 4:**
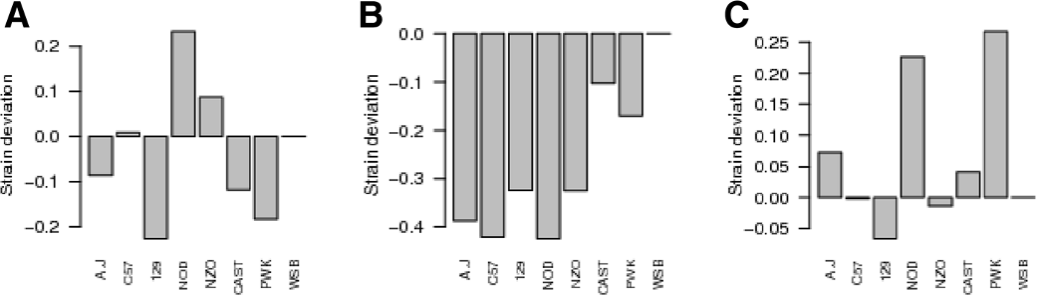
**Estimated haplotype effects at QTLs for survival time following Kp infection.** Effects are shown as deviations relative to WSB/EiJ, which is arbitrarily assigned the trait effect of 0. The x-axis of each plot shows the founder strains; the y-axis shows the estimated haplotype effects of the CC founders. **A**. *Kprl*1, **B**. *Kprl*2, **C**. *Kprl*3.

**Figure 5:**
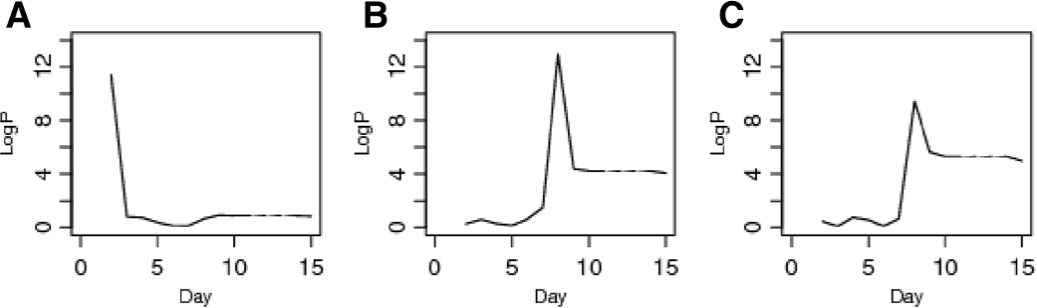
**Plots of temporal profiles of the logP at the QTLs over duration of experiment. A**. *Kprl*1 on chromosome 4, **B**. *Kprl*2 on chromosome 8 and **C**. *Kprl*3 on chromosome 18.

At each QTL we report the effect of each founder haplotype on survival time relative to WSB/EiJ, (Figure 4). All three loci showed complex patterns of haplotype effects, with the wild-derived strains playing a role but the inbred strains also contributing to the overall QTL effect. We also looked at the logP at the QTL peak at each day of the experiment and plotted the temporal profile of each QTL through the experiment (Figure 5). This showed that all three QTLs are of short duration, one at the start of the experiment and two towards the end of the progression of the disease.

**Table 1:** **Positions of QTLs associated with susceptibility to Kp infection in 48 CC lines**

QTL	Day	Chr	LogP	H ^2^	Sig	50% CI (Mb)		90% CI (Mb)		95% CI (Mb)	
						Position	Width	Position	Width	Position	Width
*Kprl*1	2	4	11.31	0.8	0.05	56.00-56.47	0.48	54.27-58.28	4.01	52.73-59.76	7.03
*Kprl*2	8	8	12.85	0.83	0.05	31.72-32.23	0.51	29.69-33.62	3.93	29.07-34.52	5.44
*Kprl*3	8	18	9.44	0.75	0.50	25.07-30.91	5.85	19.89-36.42	16.53	18.88-36.94	18.06

The chromosome (Chr), negative log_10_ p-value (logP), heritability (H^2^) and genome-wide significance level reached (Sig) are given, as well as the position and length of the 50%, 90% and 95% CIs relative to mouse genome build mm9.

### Association analysis of sequence variations and candidate genes

**Figure 6:**
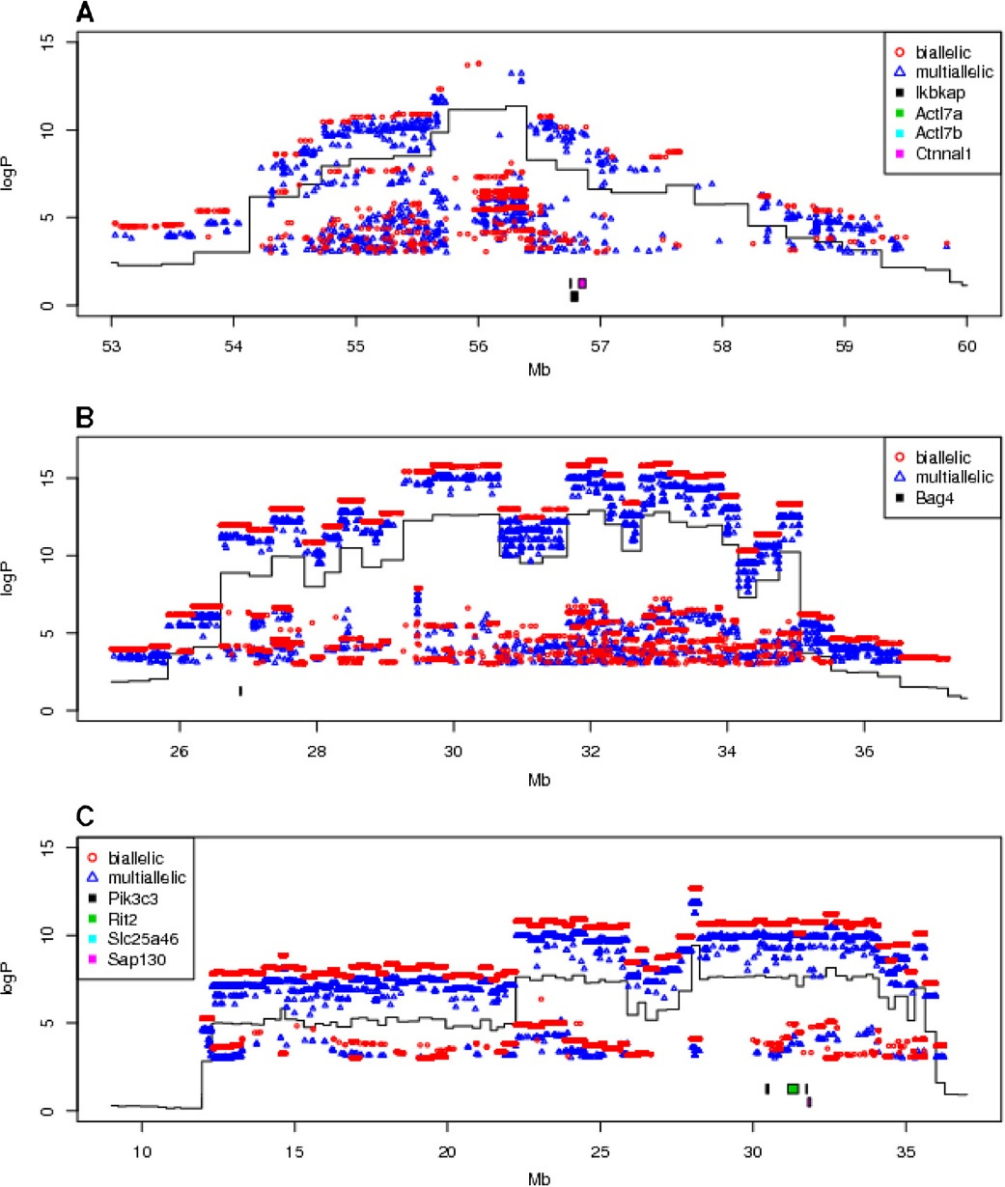
**Merge analysis of sequence variants.** The x-axis is genome location, y-axis is the logP of the test of association between locus and survival. The continuous black lines are sections of the genome scans in Figure 4. The blue dots are the results of merge analysis tests of sequence variants segregating in the 8 founders of the CC. For clarity, the great majority of variants with merge logP < 1 are not shown. Biallelic SNPs are in red circles, SNPs with more than two alleles (caused when alleles are unknown in one or more founders, which are then treated as unknown private alleles) are dark blue triangles. Image taken from the genome scan viewer http://mus.well.ox.ac.uk/gscandb. **A**. *Kprl*1 on chromosome 4; **B**. *Kprl*2 on chromosome 8 and **C**. *Kprl*3 on chromosome 18.

We used Merge Analysis [17] to impute and test association of sequence variants segregating between the CC founders within the QTLs. This takes advantage of the ancestry of the CC to infer the alleles of each CC line based on its genome mosaic (determined from its SNP genotypes) and sequence variation data in the founder strains. Where a QTL is caused by a single diallelic variant, we expect to have a high chance of testing a very tightly linked tagging SNP with the identical strain distribution pattern in the founders as the causal variant. We also expect the merge analysis of such a SNP to have a higher logP-value than the 8-way haplotype test in the interval containing the variant, due to the reduction in the dimension of the test. If this is not observed, one possibility other than a false positive is that the QTL is caused by a combination of linked variants. It is also possible that an unknown and therefore untested sequence variant (for example an indel or CNV) that is not tagged by a known SNP could be responsible, although this is unlikely since most indels have similar SDP to a neighbouring SNP, and only a limited number of distinct strain distribution patterns are ever observed in the founders at each locus [18]. The merge analysis for *Kprl*1, 2 and 3 are shown in Figure 6A, B and C respectively.

The merge analysis of the three QTLs suggested several candidate causal genes with significant merge SNPs nearby. These are listed in Additional file 4: Table S3. Consistent with our earlier findings with Aspergillosis [10], the most significant merge analysis variants involved one wild-derived strain *vs* the other 7 strains. In these QTLs we can exclude the great majority of variants from being causal. However, although the fraction of SNPs with the most associated SDP is small, these are evenly distributed across the QTL with logP values that track those of the 8-way haplotype logP. Consequently many genes under the QTL will contain or be close to a variant with that SDP. Also, these SDPs pick out only a subset of the contrasts between the 8 haplotypes that are observed at the QTL peak. At *Kprl*2 and *Kprl*3, the merge SNPs with the best logPs are biallelic, despite the haplotype effects indicating a more complex situation. Presumably, none of the multiallelic SDPs matches the true situation closely enough to overcome the dimension penalty in the test. At *Kprl*1, there are virtually no significant merge SNPs at the QTL peak and none of them have an SDP matching the haplotype effects observed at the peak.

We classified the sequence variants under the QTLs according to whether their merge logP was greater than the corresponding 8-way haplotype logP, and by their relationship to the genome annotation (Non Synonymous Coding, Synonymous Coding, 5′ and 3′ UTR, Splice Site or Intronic) (Additional file 4: Table S3).

The following genes that were identified by merge analysis are also strong candidates based on their known functions (see Additional file 4: Table S3): *Kprl*1 contained the candidates *Ikbkap* (Inhibitor of kappa light polypeptide gene enhancer in B-cells, kinase complex-associated protein). *Actl7a* and *Actl7b* (actin-like 7a and 7b), and *Ctnnal1* (catenin alpha-like 1 gene), are involved in cell adhesion and cytoskeleton structure [19, 20]. These are important in phagocytosis and for eliminating bacterial infection. *Ctnnal1* is also important in the cell adhesion process [21, 22] and for capturing bacterial molecules to eliminate infection. *Kprl*2 contains the candidate gene *Bag4* (BCL2-associated athanogene 4) [23], which mediates cell-cell and cell-extracellular matrix interactions and this complex plays a role in host response to bacterial infection [24, 25]. Homozygous mutant mice for *Bag4* have enhanced cytokine responses and increased IL-6 production following TNF challenge i.e. septic shock [23]. Finally, *Kprl*3 contains the candidate genes *Pik3c3* (phosphoinositide-3-kinase, class 3), *Rit2* (ras-like without CAAX 2), *Slc25a46* (solute carrier family 25, member 46) and *Sap130* (Sin3A associated protein). *Pik3c3* mice homozygous for a conditional allele activated in T cells exhibit impaired naive T cell homeostasis and mitophagy [26].

## Discussion

In this study, we have used collaborative cross mice to identify QTLs, and suggest candidate genes contributing to, host response to Kp infection. The identification of genetic resistant factors to this disease will help of understanding why certain hosts succumb to the infection while others do not, and this will open new avenues for developing alternative control mechanisms to the infection.

The CC lines have more variable survival times compared to classical inbred strains, presumably due to the genetic diversity of the CC founders. C3H/HeJ mouse strain which is known be TLR4 deficient [27] showed to survive longer to the infection comparing to BALB/cJ and C57BL/6 J, but similar to DBA/2 J. However, based on the published results http://www.ncbi.nlm.nih.gov/gene/21898, TLR4 locus is located on chromosome 4 at 66 Mb position, which is distal to *Kprl*1 locus, was not mapped with the tested traits and subsequently not involved or has little effect with this infection.

QTL mapping of survival times using survival regression was not successful, for despite the high heritability of survival time in the CC, no QTL was significant across all time points, indicating that the effects of any QTLs were transient. Instead, we defined a binary phenotype, alive or dead, for each day of the experiment and QTL mapping was performed separately for each day during the infection in order to detect transient QTLs. This novel analysis significantly improved power. We conclude that host susceptibility to Kp is a complex trait controlled by at least three loci acting at different times during the infection. To our knowledge, this is the first report using immuno-competent mouse strains to determine susceptibility to and map QTLs for Kp infection.

We compared these results with our previous analysis of the susceptibility of CC mice to aspergillosis [10], where the standard survival regression method, that assesses the overall effect of a locus throughout the experiment, was successful. The two infections have entirely different genetic architectures. Thus the method of analysis significantly affects the results, and the correct choice depends on the mode of action of the phenotype and possibly the immune status of the population. However, both studies show that the CC is well suited to the study of host response to infection, presumably because of the accumulation of genetic variation private to different subspecies, which presumably were exposed to differential selection pressures. Using the full genome sequence data of the eight founders of the CC [7], with the advantage of the merge analysis [17], it possible to refine the QTL map interval to small genomic regions and identify strong candidate genes. Searching for candidate genes within the fine mapped QTL regions revealed genes involve in T-cells regulations. One of the interesting candidate genes was found within *Kprl*2 QTL, *Bag4* (BCL2-associated athanogene 4) [23], which mediates cell-cell and cell-extracellular matrix interactions and this complex plays a role in host response to bacterial infection [24, 25]. Based on previous research, it was shown that homozygous mutant mice for *Bag4* have enhanced cytokine responses and increased IL-6 production following TNF challenge i.e. septic shock [23] (Kp is a major pathogenic cause of sepsis).

Identifying QTL act at different time points during challenge i.e. early v.s late phase was reported, earlier with trypanosome infection [28]. This is not surprising machines knowing that the host innate and adaptive immunity systems act at different time points during infection.

This report and those in [29–33] demonstrate the utility of the CC in the analysis of complex traits in mouse models of human disease. Our results underline the importance of the contribution of wild-derived alleles to the CC: the majority of the QTL we mapped involved a contrast with one of the three wild strains. The wild-derived founders may have different immune response mechanisms compared with the classical mouse strains. If so, we expect to identify novel response mechanisms to infectious diseases. This has two consequences. First, we can identify more genes using populations in which these variants segregate than in classical populations. Second, only sequence variants segregating in the CC founders that follow the same strain distribution pattern can be causal for the QTL. Therefore having a complete catalogue of sequence variants in the founders is of great utility. In effect, the combination of high-density genotypes in the CC and the genome sequences of the CC founders yield an approximate reconstruction of the sequence of each CC line as a mosaic of fragments of the founders’ genomes. While this is currently limited to those regions of the genome that can be assembled from short-read sequence data, as sequencing technologies improve we expect to generate a complete catalogue of variation in the CC.

## Conclusion

Our study has shown that host susceptibility to Kp is a complex trait, controlled by multiple genetic factors that act sequentially during the course of infection. The current study shows that even a modest number of lines [17] are useful with sufficient replication within each line. Nonetheless, in the near future over a hundred inbred and genotyped CC lines will be available to the research community, and using more lines will improve mapping resolution and power.

## Electronic supplementary material

Additional file 1: Table S1: Mean and st. dev. of survival times of four inbred strains, DBA/2J, C3H/HeJ, C57BL/6J and BALB/CJ, and p-values of T-tests of comparisons between BALB/cJ and the other strains. (DOC 30 KB)

Additional file 2: Table S2: Mean survival time in days of the different collaborative cross (CC) lines following infection with *Klebsiella Pneumonia*. Std error of survival time in days for each line is presented. (DOC 119 KB)

Additional file 3: Figure S1: Summary plots for simulations conducted to calculate confidence intervals (CIs) for the QTLs. (DOC 353 KB)

Additional file 4: Table S3: A. Locus *Kprl*1: Significant merge SNPs in genes in the 50%, 90% and 95% Confidence Intervals, and their functional consequence. Significant merge SNPs are defined as SNPs with a logP greater than the logP for the haplotype test. Candidate genes are in bold type. **Table S3.** B. Locus *Kprl*2: Significant merge SNPs in genes in the 50%, 90% and 95% Confidence Intervals, and their functional consequence. Significant merge SNPs are defined as SNPs with a logP greater than the Additional file 3: e logP for the haplotype test. **Table S3.** C. Locus *Kprl*3: Significant merge SNPs in genes in the 50%, 90% and 95% Confidence Intervals, and their functional consequence. Significant merge SNPs are defined as SNPs with a logP greater than the logP for the haplotype test. Candidate genes are in bold type. (ZIP 179 KB)

## Abbreviations

CC: Collaborative cross
CTC: Complex trait consortium
QTL: Quantitative trait loci
TAU: Tel Aviv University
Kp: Klebsiella pneumonaie
Kprl: ***K***lebsiella ***p***neumonia ***r***esistant ***l***ocus
CIs: confidence intervals
SNP: single nucleotide polymorphic
WTCHG: Wellcome Trsust Center for Human Genetics
UNC: University of North Carolina
cfu: Colony forming units.
