# Supplementary material for: Susceptibility to klebsiella pneumonaie infection in collaborative cross mice is a complex trait controlled by at least three loci acting at different time points
Source: BMC Genomics. 2014 Oct 6;15(1):865. doi: 10.1186/1471-2164-15-865 (PMC4201739; doi:10.1186/1471-2164-15-865)
Supplement: Supplementary file 1 — Additional file 1: Table S1: Mean and st. dev. of survival times of four inbred strains, DBA/2J, C3H/HeJ, C57BL/6J and BALB/CJ, and p-values of T-tests of comparisons between BALB/cJ and the other strains. (DOC 30 KB) [file 12864_2014_6555_MOESM1_ESM.doc]

**Supplemental Table S1. Mean and st. dev. of survival times of four inbred strains, DBA/2J, C3H/HeJ, C57BL/6J and BALB/CJ, and p-values of T-tests of comparisons between BALB/cJ and the other strains.**

|  | **Mean survival time**  **(days)** | **St. dev. of survival time**  **(days)** | **Difference from BALB/cJ**  **(p-value)** |
| --- | --- | --- | --- |
| **BALB/cJ** | 2.0 | 0.6 | NA |
| **DBA/2J** | 4.0 | 1.88 | 0.004 |
| **C3H/HeJ** | 3.8 | 1.16 | 0.001 |
| **C57BL/6J** | 2.8 | 0.56 | 0.029 |
