# Supplementary material for: Susceptibility to klebsiella pneumonaie infection in collaborative cross mice is a complex trait controlled by at least three loci acting at different time points
Source: BMC Genomics. 2014 Oct 6;15(1):865. doi: 10.1186/1471-2164-15-865 (PMC4201739; doi:10.1186/1471-2164-15-865)
Supplement: Supplementary file 2 — Additional file 2: Table S2: Mean survival time in days of the different collaborative cross (CC) lines following infection with Klebsiella Pneumonia. Std error of survival time in days for each line is presented. (DOC 119 KB) [file 12864_2014_6555_MOESM2_ESM.doc]

**Supplemental Table S2.** Mean survival time in days of the different collaborative cross (CC) lines following infection with *Klebsiella Pneumonia*. Std error of survival time in days for each line is presented.

|  | CC line | Number of mice | Mean survival | Std. Deviation | Std. Error |
| --- | --- | --- | --- | --- | --- |
|  |  |  |  |  |  |
| 1 | IL-7 | 4 | 4 | 2.309 | 1.155 |
| 2 | IL-11 | 2 | 5.5 | 2.121 | 1.5 |
| 3 | IL-19 | 5 | 2.8 | 1.924 | 0.86 |
| 4 | IL-21 | 7 | 2.29 | 1.604 | 0.606 |
| 5 | IL-24 | 4 | 2.25 | 1.893 | 0.946 |
| 6 | IL-26 | 8 | 3.75 | 1.488 | 0.526 |
| 7 | IL-27 | 7 | 3 | 1.155 | 0.436 |
| 8 | IL-30 | 4 | 8.75 | 7.228 | 3.614 |
| 9 | IL-33 | 2 | 1 | 0 | 0 |
| 10 | IL-34 | 6 | 5 | 0 | 0 |
| 11 | IL-50 | 2 | 1 | 0 | 0 |
| 12 | IL-72 | 15 | 5.4 | 4.323 | 1.116 |
| 13 | IL-76 | 3 | 3.67 | 2.309 | 1.333 |
| 14 | IL-96 | 4 | 3 | 1.633 | 0.816 |
| 15 | IL-100 | 2 | 8.5 | 9.192 | 6.5 |
| 16 | IL-104 | 4 | 2.75 | 2.217 | 1.109 |
| 17 | IL-111 | 7 | 3.86 | 0.9 | 0.34 |
| 18 | IL-114 | 2 | 7.5 | 0.707 | 0.5 |
| 19 | IL-134 | 8 | 2.88 | 1.959 | 0.693 |
| 20 | IL-135 | 4 | 9.5 | 6.351 | 3.175 |
| 21 | IL-140 | 10 | 3.9 | 2.183 | 0.69 |
| 22 | IL-143 | 5 | 6.8 | 4.919 | 2.2 |
| 23 | IL-145 | 7 | 3.86 | 2.116 | 0.8 |
| 24 | IL-150 | 2 | 6 | 0 | 0 |
| 25 | IL-166 | 4 | 2 | 0.816 | 0.408 |
| 26 | IL-179 | 3 | 2 | 0 | 0 |
| 27 | IL-182 | 8 | 8.88 | 5.167 | 1.827 |
| 28 | IL-188 | 2 | 3.5 | 0.707 | 0.5 |
| 29 | IL-196 | 11 | 7.73 | 5.711 | 1.722 |
| 30 | IL-199 | 3 | 1.67 | 1.155 | 0.667 |
| 31 | IL-211 | 8 | 3.5 | 2.07 | 0.732 |
| 32 | IL-217 | 4 | 2 | 0.816 | 0.408 |
| 33 | IL-219 | 7 | 3.43 | 1.813 | 0.685 |
| 34 | IL-285 | 7 | 2.71 | 0.488 | 0.184 |
| 35 | IL-287 | 1 | 5 | . | . |
| 36 | IL-299 | 2 | 2.5 | 0.707 | 0.5 |
| 37 | IL-312 | 3 | 2 | 0 | 0 |
| 38 | IL-316 | 2 | 7 | 2.828 | 2 |
| 39 | IL-511 | 3 | 4 | 1.732 | 1 |
| 40 | IL-515 | 3 | 4.33 | 2.309 | 1.333 |
| 41 | IL-518 | 4 | 4 | 1.155 | 0.577 |
| 42 | IL-519 | 3 | 2 | 0 | 0 |
| 43 | IL-530 | 1 | 6 | . | . |
| 44 | IL-534 | 7 | 4.14 | 1.952 | 0.738 |
| 45 | IL-540 | 9 | 3 | 0 | 0 |
| 46 | IL-549 | 3 | 5.33 | 0.577 | 0.333 |
| 47 | IL-550 | 4 | 2.75 | 1.258 | 0.629 |
| 48 | IL-551 | 6 | 5.5 | 4.68 | 1.91 |
| 49 | IL-555 | 3 | 2.67 | 1.528 | 0.882 |
| 50 | IL-557 | 7 | 3.29 | 1.254 | 0.474 |
| 51 | IL-568 | 2 | 3.5 | 0.707 | 0.5 |
| 52 | IL-572 | 5 | 2 | 0 | 0 |
| 53 | IL-577 | 3 | 1.67 | 1.155 | 0.667 |
| 54 | IL-599 | 3 | 12 | 5.196 | 3 |
| 55 | IL-608 | 3 | 5 | 1.732 | 1 |
| 56 | IL-611 | 6 | 3.5 | 1.975 | 0.806 |
| 57 | IL-621 | 4 | 5.75 | 1.258 | 0.629 |
| 58 | IL-627 | 4 | 5 | 2 | 1 |
| 59 | IL-633 | 6 | 7.33 | 5.955 | 2.431 |
| 60 | IL-635 | 5 | 7.6 | 6.768 | 3.027 |
| 61 | IL-643 | 2 | 4 | 2.828 | 2 |
| 62 | IL-645 | 1 | 2 | . | . |
| 63 | IL-666 | 4 | 5.25 | 1.5 | 0.75 |
| 64 | IL-699 | 3 | 7.33 | 6.658 | 3.844 |
| 65 | IL-703 | 2 | 2 | 0 | 0 |
| 66 | IL-711 | 2 | 2.5 | 0.707 | 0.5 |
| 67 | IL-719 | 5 | 2.8 | 0.447 | 0.2 |
| 68 | IL-785 | 3 | 6 | 0 | 0 |
| 69 | IL-787 | 4 | 5 | 0 | 0 |
| 70 | IL-811 | 4 | 6 | 6 | 3 |
| 71 | IL-812 | 5 | 3.4 | 0.894 | 0.4 |
| 72 | IL-813 | 8 | 2.5 | 0.926 | 0.327 |
| 73 | IL-817 | 2 | 4.5 | 2.121 | 1.5 |
|  | total | 328 |  |  |  |
