# Supplementary material for: Susceptibility to klebsiella pneumonaie infection in collaborative cross mice is a complex trait controlled by at least three loci acting at different time points
Source: BMC Genomics. 2014 Oct 6;15(1):865. doi: 10.1186/1471-2164-15-865 (PMC4201739; doi:10.1186/1471-2164-15-865)

Supplemental Figure S1. Summary plots for simulations conducted to calculate confidence intervals (CIs) for the QTLs. QTLs with the same effect parameters as the original detected QTL were simulated at locations around the original detected location. For each simulated phenotype at each simulated location, QTL mapping was conducted across the region and the maximum logP in the region and its location were recorded. The mapping distance error was defined as the distance between the simulated location of the QTL and the location of the maximum logP in the region from QTL mapping with the simulated phenotype. This was measured in both Mb and in the number of marker intervals.

Locus *Kprl*1: **Top left**: Histogram of the mapping distance error in Mb.
Top right: Box-and-whisker plot showing the range of values observed for the maximum logP over simulations with equal mapping distance error for each value of the mapping distance error measured in marker intervals.
Middle left: Cumulative distribution of the absolute value of the mapping distance error.
Middle right: Histogram of the marker intervals in the region around the original detected QTL selected for the 100 simulated locations.
Bottom left: Box-and-whisker plot of the range of mapped locations (marker intervals) for each simulated location (marker interval).
Bottom right: Box-and-whisker plot of the range of values of the mapping distance error (in Mb) for each simulated location (marker interval).

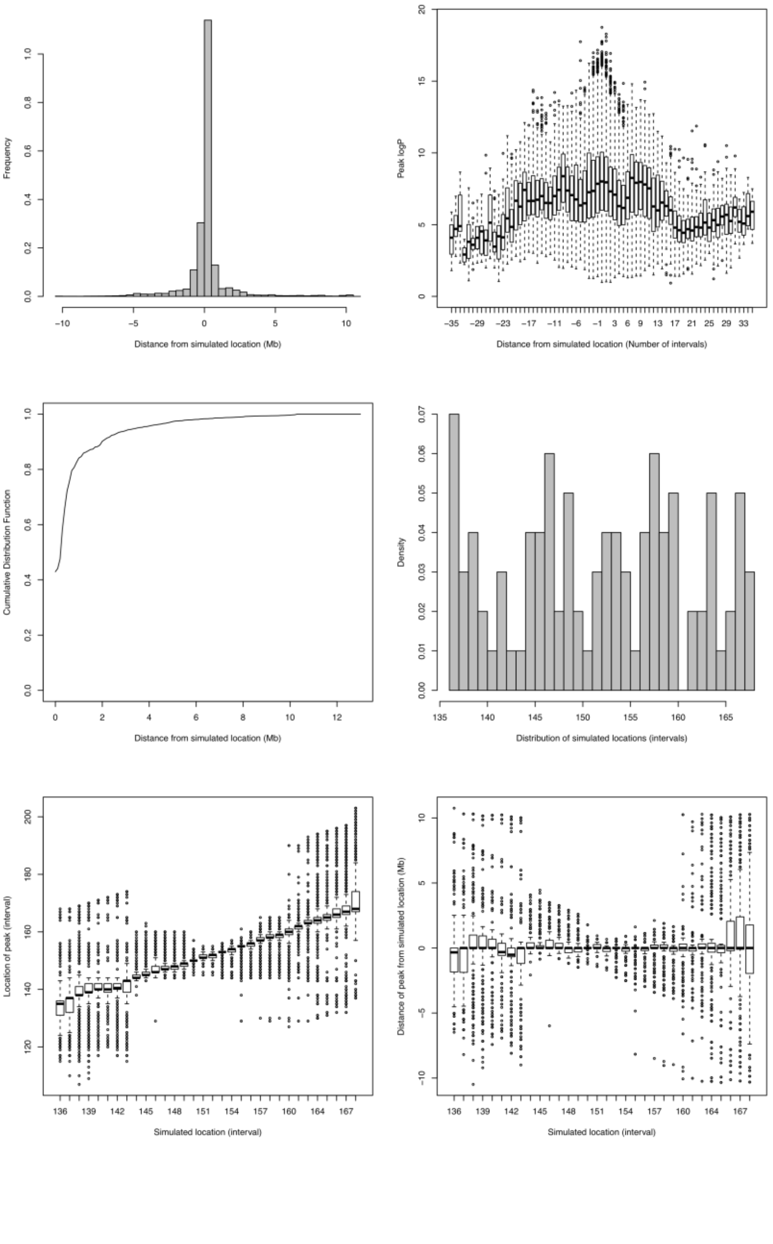

Locus *Kprl2*: Top left: Histogram of the mapping distance error in Mb.
Top right: Box-and-whisker plot showing the range of values observed for the maximum logP over simulations with equal mapping distance error for each value of the mapping distance error measured in marker intervals.
Middle left: Cumulative distribution of the absolute value of the mapping distance error.
Middle right: Histogram of the marker intervals in the region around the original detected QTL selected for the 100 simulated locations.
Bottom left: Box-and-whisker plot of the range of mapped locations (marker intervals) for each simulated location (marker interval).
Bottom right: Box-and-whisker plot of the range of values of the mapping distance error (in Mb) for each simulated location (marker interval).

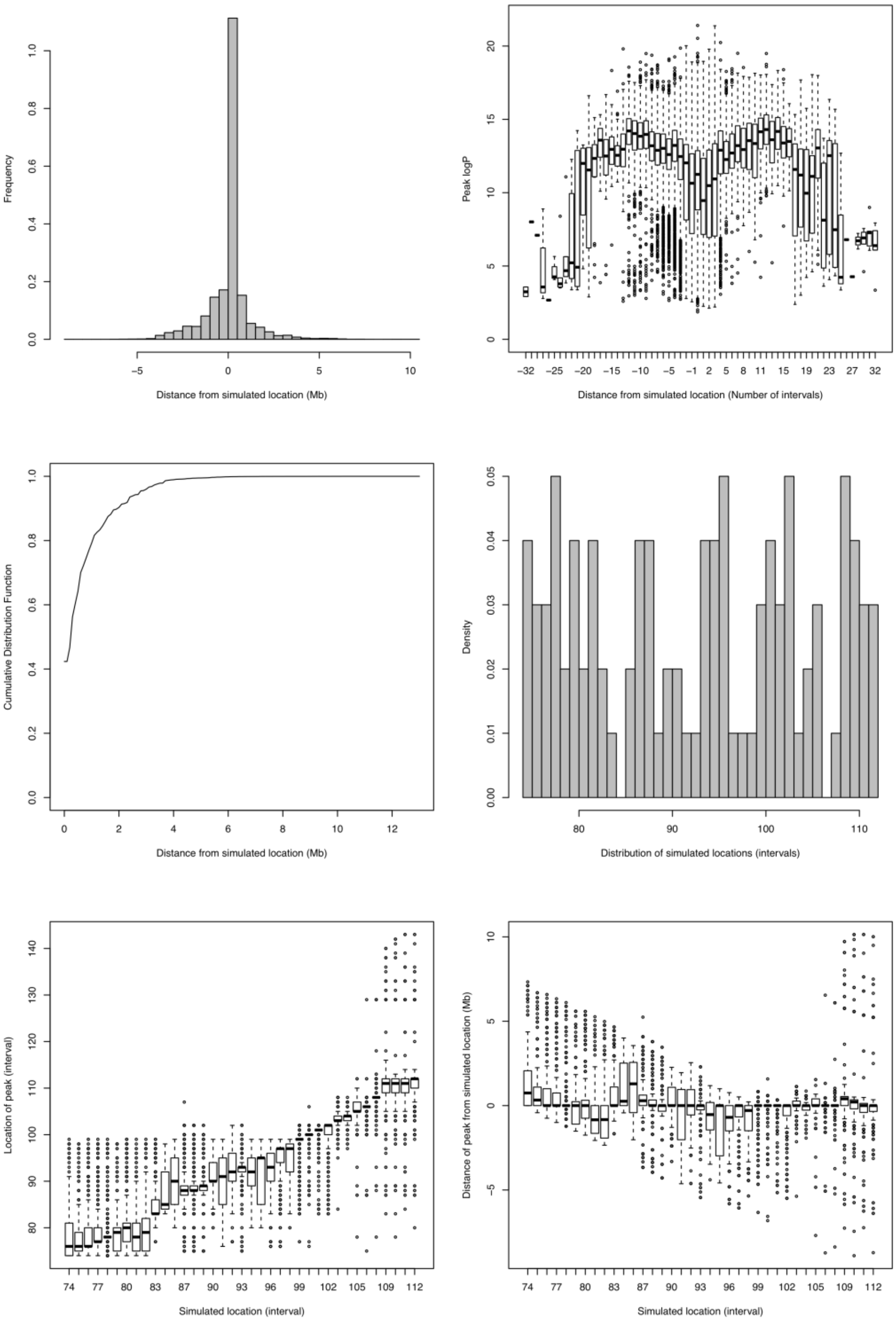

Locus *Kprl*3: Top left: Histogram of the mapping distance error in Mb.
Top right: Box-and-whisker plot showing the range of values observed for the maximum logP over simulations with equal mapping distance error for each value of the mapping distance error measured in marker intervals.
Middle left: Cumulative distribution of the absolute value of the mapping distance error.
Middle right: Histogram of the marker intervals in the region around the original detected QTL selected for the 100 simulated locations.
Bottom left: Box-and-whisker plot of the range of mapped locations (marker intervals) for each simulated location (marker interval).
Bottom right: Box-and-whisker plot of the range of values of the mapping distance error (in Mb) for each simulated location (marker interval).

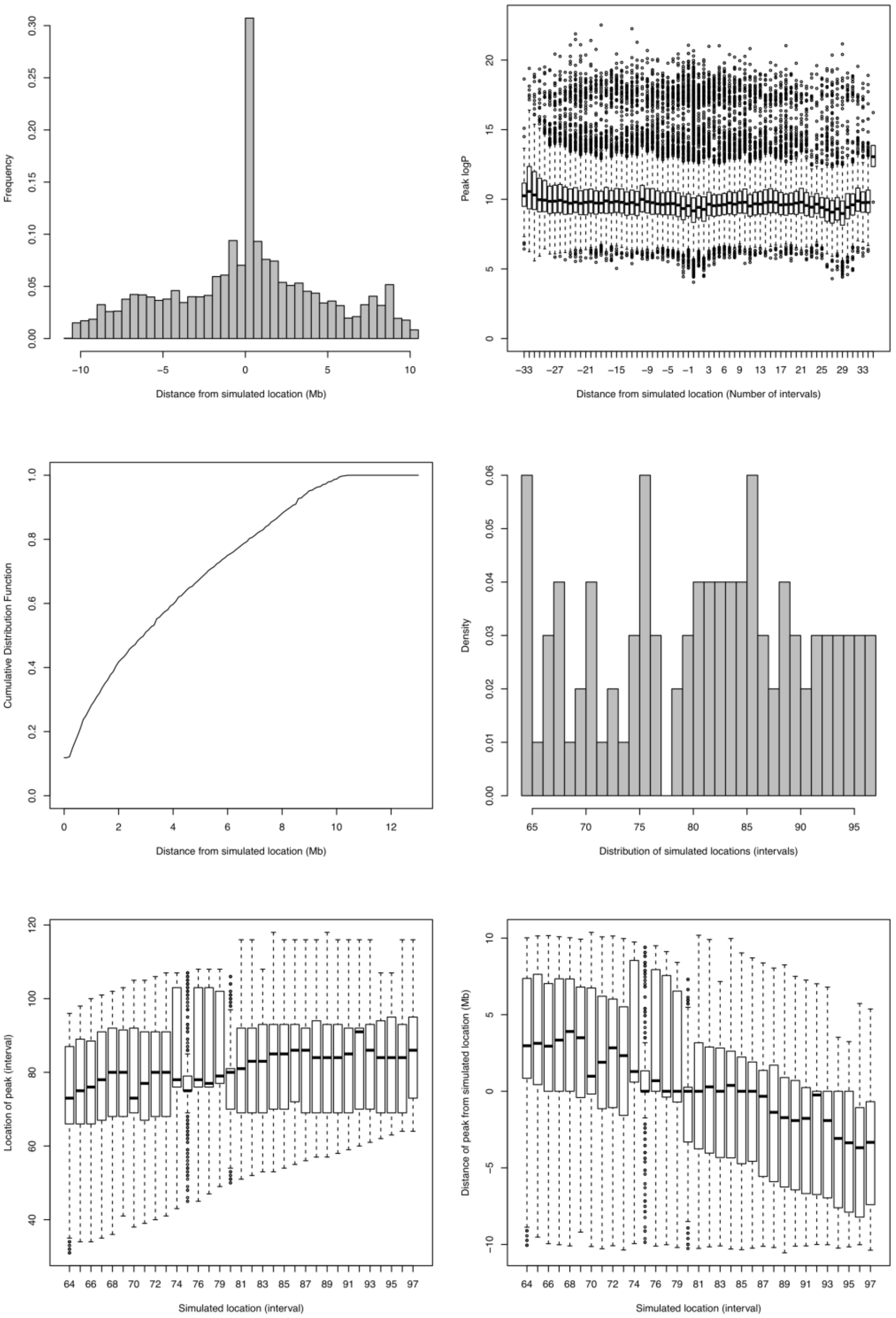

Supplement: Supplementary file 3 — Additional file 3: Figure S1: Summary plots for simulations conducted to calculate confidence intervals (CIs) for the QTLs. (DOC 353 KB) [file 12864_2014_6555_MOESM3_ESM.doc]
