# Supplementary material for: Susceptibility to klebsiella pneumonaie infection in collaborative cross mice is a complex trait controlled by at least three loci acting at different time points
Source: BMC Genomics. 2014 Oct 6;15(1):865. doi: 10.1186/1471-2164-15-865 (PMC4201739; doi:10.1186/1471-2164-15-865)
Supplement: Supplementary file 4 — Additional file 4: Table S3: A. Locus Kprl1: Significant merge SNPs in genes in the 50%, 90% and 95% Confidence Intervals, and their functional consequence. Significant merge SNPs are defined as SNPs with a logP greater than the logP for the haplotype test. Candidate genes are in bold type. Table S3. B. Locus Kprl2: Significant merge SNPs in genes in the 50%, 90% and 95% Confidence Intervals, and their functional consequence. Significant merge SNPs are defined as SNPs with a logP greater than the Additional file 3: e logP for the haplotype test. Table S3. C. Locus Kprl3: Significant merge SNPs in genes in the 50%, 90% and 95% Confidence Intervals, and their functional consequence. Significant merge SNPs are defined as SNPs with a logP greater than the logP for the haplotype test. Candidate genes are in bold type. (ZIP 179 KB) [file 12864_2014_6555_MOESM4_ESM.zip › add3/1340885795128759_add1a.docx]

**Table ST3: A. Locus *Kprl*1**: Significant merge SNPs in genes in the 50%, 90% and 95% Confidence Intervals, and their functional consequence. Significant merge SNPs are defined as SNPs with a logP greater than the logP for the haplotype test. Candidate genes are in bold type.

| % CI | Gene Name | N Sig SNPs | INTRONIC | 3’ UTR | 5’ UTR | SYNONYMOUS CODING | NON SYNONYMOUS CODING |
| --- | --- | --- | --- | --- | --- | --- | --- |
| 90 | *6430704M03Rik* | 4 | 4 |  |  |  |  |
| **90** | ***Ctnnal1*** | **2** | **2** |  |  |  |  |
| 90 | *Ptpn3* | 2 | 2 |  |  |  |  |
| 90 | *Zfp462* | 16 | 13 | 3 |  |  |  |
| 90 | *Rad23b* | 6 | 6 |  |  |  |  |
| 90 | *AL732494.16* | 10 | 10 |  |  |  |  |
| **90** | ***Ikbkap*** | **6** | **6** |  |  |  |  |
| 90 | *Epb4.1l4b* | 18 | 18 |  |  |  |  |
| 90 | *D730040F13Rik* | 4 |  | 4 |  |  |  |
| 90 | *Palm2* | 18 | 18 |  |  |  |  |
|  |  |  |  |  |  |  |  |
| 95 | *AL807745.7-2* | 1 | 1 |  |  |  |  |
| 95 | *Nipsnap3a* | 1 | 1 |  |  |  |  |
| 95 | *Dnajc25* | 2 | 2 |  |  |  |  |
| 95 | *AI481877* | 8 | 8 |  |  |  |  |
| 95 | *Musk* | 14 | 14 |  |  |  |  |
| 95 | *Ugcg* | 1 | 1 |  |  |  |  |
| 95 | *Olfr272* | 34 | 32 |  | 2 |  |  |
| 95 | *Tmem38b* | 2 | 2 |  |  |  |  |
| 95 | *Abca1* | 12 | 12 |  |  |  |  |
| 95 | *AI314180* | 19 | 19 |  |  |  |  |
| 95 | *Fsd1l* | 1 | 1 |  |  |  |  |
| 95 | *Fktn* | 2 | 2 |  |  |  |  |
| 95 | *Olfr275* | 3 |  |  |  | 2 | 1 |
| 95 | *Tal2* | 1 | 1 |  |  |  |  |
| 95 | *AI427809* | 1 | 1 |  |  |  |  |
| 95 | *Slc44a1* | 110 | 110 |  |  |  |  |
| 95 | *Zkscan16* | 2 | 2 |  |  |  |  |
| 95 | *Lpar1* | 8 | 8 |  |  |  |  |
| 95 | *Ptgr1* | 1 | 1 |  |  |  |  |
