# Supplementary material for: Susceptibility to klebsiella pneumonaie infection in collaborative cross mice is a complex trait controlled by at least three loci acting at different time points
Source: BMC Genomics. 2014 Oct 6;15(1):865. doi: 10.1186/1471-2164-15-865 (PMC4201739; doi:10.1186/1471-2164-15-865)
Supplement: Supplementary file 4 — Additional file 4: Table S3: A. Locus Kprl1: Significant merge SNPs in genes in the 50%, 90% and 95% Confidence Intervals, and their functional consequence. Significant merge SNPs are defined as SNPs with a logP greater than the logP for the haplotype test. Candidate genes are in bold type. Table S3. B. Locus Kprl2: Significant merge SNPs in genes in the 50%, 90% and 95% Confidence Intervals, and their functional consequence. Significant merge SNPs are defined as SNPs with a logP greater than the Additional file 3: e logP for the haplotype test. Table S3. C. Locus Kprl3: Significant merge SNPs in genes in the 50%, 90% and 95% Confidence Intervals, and their functional consequence. Significant merge SNPs are defined as SNPs with a logP greater than the logP for the haplotype test. Candidate genes are in bold type. (ZIP 179 KB) [file 12864_2014_6555_MOESM4_ESM.zip › add3/1340885795128759_add1b.docx]

**Table ST3: B. Locus *Kprl*2**: Significant merge SNPs in genes in the 50%, 90% and 95% Confidence Intervals, and their functional consequence. Significant merge SNPs are defined as SNPs with a logP greater than the logP for the haplotype test.

| % CI | Gene Name | N Sig SNPs | INTRONIC | 3’ UTR | SYNONYMOUS CODING | | |
| --- | --- | --- | --- | --- | --- | --- | --- |
|  |  |  |  |  |  | INTRONIC | 3’ UTR |
| 50 | *Rnf122* | 2 | 2 |  |  |  |  |
| 50 | *Dusp26* | 30 | 29 |  | 1 |  |  |
|  |  |  |  |  |  |  |  |
| 90 | *Nrg1* | 253 | 251 |  |  | 2 |  |
| 90 | *Fut10* | 175 | 173 | 2 |  |  |  |
| 90 | *Mak16* | 2 | 2 |  |  |  |  |
| 90 | *Unc5d* | 413 | 412 |  | 1 |  |  |
|  |  |  |  |  |  |  |  |
| 95 | *100042715-201* | 182 | 182 |  |  |  |  |
| 95 | *Purg* | 11 | 11 |  |  |  |  |
| 95 | *Wrn* | 164 | 161 |  | 1 | 1 | 1 |
