# Supplementary material for: Susceptibility to klebsiella pneumonaie infection in collaborative cross mice is a complex trait controlled by at least three loci acting at different time points
Source: BMC Genomics. 2014 Oct 6;15(1):865. doi: 10.1186/1471-2164-15-865 (PMC4201739; doi:10.1186/1471-2164-15-865)
Supplement: Supplementary file 4 — Additional file 4: Table S3: A. Locus Kprl1: Significant merge SNPs in genes in the 50%, 90% and 95% Confidence Intervals, and their functional consequence. Significant merge SNPs are defined as SNPs with a logP greater than the logP for the haplotype test. Candidate genes are in bold type. Table S3. B. Locus Kprl2: Significant merge SNPs in genes in the 50%, 90% and 95% Confidence Intervals, and their functional consequence. Significant merge SNPs are defined as SNPs with a logP greater than the Additional file 3: e logP for the haplotype test. Table S3. C. Locus Kprl3: Significant merge SNPs in genes in the 50%, 90% and 95% Confidence Intervals, and their functional consequence. Significant merge SNPs are defined as SNPs with a logP greater than the logP for the haplotype test. Candidate genes are in bold type. (ZIP 179 KB) [file 12864_2014_6555_MOESM4_ESM.zip › add3/1340885795128759_add1c.docx]

**Table ST3: C. Locus *Kprl*3**: Significant merge SNPs in genes in the 50%, 90% and 95% Confidence Intervals, and their functional consequence. Significant merge SNPs are defined as SNPs with a logP greater than the logP for the haplotype test. Candidate genes are in bold type.

| %CI | Gene Name | N Sig SNPs | Intronic | | 5’ UTR | 3’ UTR | Synonymous Coding | | | | Non- Synonymous Coding | | Splice Site | | | |
| --- | --- | --- | --- | --- | --- | --- | --- | --- | --- | --- | --- | --- | --- | --- | --- | --- |
|  |  |  |  | 3’ UTR |  |  |  | Intronic | | Splice Site |  | Intronic |  | Intronic | 5’ UTR | Non- Synonymous Coding |
|  |  |  |  |  |  |  |  |  | 3’ UTR |  |  |  |  |  |  |  |
| **50** | ***Pik3c3*** | **537** | **520** | **6** |  |  | **7** |  |  |  | **2** |  |  | **2** |  |  |
|  |  |  |  |  |  |  |  |  |  |  |  |  |  |  |  |  |
| 90 | *Kif20a* | 15 | 10 |  |  |  | 3 |  |  |  | 2 |  |  |  |  |  |
| 90 | *4933408B17Rik* | 49 | 46 |  | 3 |  |  |  |  |  |  |  |  |  |  |  |
| 90 | *Dnajc18* | 150 | 150 |  |  |  |  |  |  |  |  |  |  |  |  |  |
| 90 | *Pkd2l2* | 120 | 111 |  |  |  | 8 |  |  |  | 1 |  |  |  |  |  |
| 90 | *Reep5* | 151 | 140 |  |  | 11 |  |  |  |  |  |  |  |  |  |  |
| 90 | *Paip2* | 24 | 24 |  |  |  |  |  |  |  |  |  |  |  |  |  |
| 90 | *Srp19* | 8 | 7 |  | 1 |  |  |  |  |  |  |  |  |  |  |  |
| 90 | *Ammecr1l* | 36 | 32 |  |  | 3 | 1 |  |  |  |  |  |  |  |  |  |
| 90 | *2010001M09Rik* | 7 | 6 |  |  |  | 1 |  |  |  |  |  |  |  |  |  |
| 90 | *2810012G03Rik* | 33 | 20 |  |  | 11 | 1 |  |  |  | 1 |  |  |  |  |  |
| 90 | *Jmjd1b* | 213 | 200 | 4 | 1 | 2 | 1 | 3 | 2 |  |  |  |  |  |  |  |
| 90 | *Stard4* | 19 | 18 |  |  | 1 |  |  |  |  |  |  |  |  |  |  |
| 90 | *Matr3* | 26 | 25 |  |  |  | 1 |  |  |  |  |  |  |  |  |  |
| 90 | *Camk4* | 208 | 206 |  | 1 | 1 |  |  |  |  |  |  |  |  |  |  |
| 90 | *Gm1614* | 6 |  |  |  |  | 3 |  |  |  | 3 |  |  |  |  |  |
| 90 | *Apc* | 441 | 412 |  |  | 4 | 15 | 1 |  |  | 8 |  |  | 1 |  |  |
| 90 | *Sil1* | 704 | 702 |  |  |  | 2 |  |  |  |  |  |  |  |  |  |
| 90 | *Cdc25c* | 114 | 102 |  | 3 | 4 | 3 |  |  |  | 2 |  |  |  |  |  |
| 90 | *Cdc23* | 52 | 50 |  |  |  | 1 |  |  |  | 1 |  |  |  |  |  |
| 90 | *AC115117.5* | 6 | 4 |  |  | 2 |  |  |  |  |  |  |  |  |  |  |
| 90 | *AC098881.3* | 129 | 120 |  | 1 | 7 |  |  |  |  |  |  |  |  | 1 |  |
| 90 | *Nme5* | 61 | 61 |  |  |  |  |  |  |  |  |  |  |  |  |  |
| 90 | *Slc23a1* | 18 | 15 |  |  | 2 | 1 |  |  |  |  |  |  |  |  |  |
| **90** | ***Sap130*** | **233** | **227** |  |  | **1** | **4** |  |  |  | **1** |  |  |  |  |  |
| 90 | *Wdr33* | 322 | 320 |  |  |  | 2 |  |  |  |  |  |  |  |  |  |
| 90 | *Polr2d* | 16 | 16 |  |  |  |  |  |  |  |  |  |  |  |  |  |
| 90 | *Epb4.1l4a* | 1052 | 1041 |  | 1 | 2 | 5 |  |  |  | 2 |  |  | 1 |  |  |
| 90 | *Wnt8a* | 14 | 13 |  |  | 1 |  |  |  |  |  |  |  |  |  |  |
| 90 | *Wdr36* | 159 | 153 |  |  |  | 5 |  |  |  | 1 |  |  |  |  |  |
| **90** | ***Slc25a46*** | **202** | **188** |  |  | **11** | **2** |  |  |  |  | **1** |  |  |  |  |
| 90 | *Ecscr* | 48 | 39 |  | 1 | 3 | 1 |  |  | 1 | 3 |  |  |  |  |  |
| 90 | *Gfra3* | 103 | 94 |  | 1 | 3 | 3 |  |  |  | 1 |  |  | 1 |  |  |
| 90 | *Bin1* | 172 | 169 |  |  | 2 | 1 |  |  |  |  |  |  |  |  |  |
| 90 | *Fam13b* | 133 | 131 |  |  |  |  |  |  |  | 2 |  |  |  |  |  |
| 90 | *Ctnna1* | 152 | 146 |  |  | 4 | 2 |  |  |  |  |  |  |  |  |  |
| 90 | *Etf1* | 67 | 67 |  |  |  |  |  |  |  |  |  |  |  |  |  |
| 90 | *Ercc3* | 115 | 111 |  |  |  | 2 |  |  |  |  |  |  | 2 |  |  |
| 90 | *1700066B19Rik* | 15 | 2 |  |  | 13 |  |  |  |  |  |  |  |  |  |  |
| 90 | *Tslp* | 3 | 3 |  |  |  |  |  |  |  |  |  |  |  |  |  |
| 90 | *Map3k2* | 258 | 257 |  |  |  |  |  |  |  |  |  |  | 1 |  |  |
| 90 | *Tmem173* | 14 | 11 |  |  | 1 |  |  |  |  | 2 |  |  |  |  |  |
| 90 | *Hspa9* | 168 | 162 |  |  | 3 | 2 |  |  | 1 |  |  |  |  |  |  |
| 90 | *Iws1* | 59 | 56 |  | 2 | 1 |  |  |  |  |  |  |  |  |  |  |
| 90 | *5133400G04Rik* | 8 | 8 |  |  |  |  |  |  |  |  |  |  |  |  |  |
| 90 | *Lims2* | 62 | 59 |  |  | 2 | 1 |  |  |  |  |  |  |  |  |  |
| 90 | *Reep2* | 54 | 35 |  |  | 12 | 5 |  |  | 1 |  |  |  | 1 |  |  |
| 90 | *Brd8* | 60 | 52 |  | 1 | 1 | 3 |  |  |  | 1 | 1 |  | 1 |  |  |
| 90 | *Syt4* | 13 | 7 |  | 2 | 4 |  |  |  |  |  |  |  |  |  |  |
| **90** | ***Rit2*** | **1370** | **1364** |  |  | **6** |  |  |  |  |  |  |  |  |  |  |
| 90 | *D0H4S114* | 40 | 40 |  |  |  |  |  |  |  |  |  |  |  |  |  |
| 90 | *Myo7b* | 427 | 408 |  |  |  | 16 |  |  |  | 2 |  |  |  |  | 1 |
| 90 | *Egr1* | 9 | 2 |  |  | 4 | 3 |  |  |  |  |  |  |  |  |  |
| 90 | *Proc* | 59 | 58 |  |  |  | 1 |  |  |  |  |  |  |  |  |  |
